# Assessing the Efficiency of Phenotyping Early Traits in a Greenhouse Automated Platform for Predicting Drought Tolerance of Soybean in the Field

**DOI:** 10.3389/fpls.2018.00587

**Published:** 2018-05-03

**Authors:** Laura S. Peirone, Gustavo A. Pereyra Irujo, Alejandro Bolton, Ignacio Erreguerena, Luis A. N. Aguirrezábal

**Affiliations:** ^1^Laboratorio de Fisiología Vegetal, Facultad de Ciencias Agrarias, Universidad Nacional de Mar del Plata, Balcarce, Argentina; ^2^Consejo Nacional de Investigaciones Científicas y Técnicas, Buenos Aires, Argentina; ^3^Agronomy Department, Instituto Nacional de Tecnología Agropecuaria, Balcarce, Argentina

**Keywords:** phenotyping, drought susceptibility index, transpiration efficiency, soybean, field

## Abstract

Conventional field phenotyping for drought tolerance, the most important factor limiting yield at a global scale, is labor-intensive and time-consuming. Automated greenhouse platforms can increase the precision and throughput of plant phenotyping and contribute to a faster release of drought tolerant varieties. The aim of this work was to establish a framework of analysis to identify early traits which could be efficiently measured in a greenhouse automated phenotyping platform, for predicting the drought tolerance of field grown soybean genotypes. A group of genotypes was evaluated, which showed variation in their drought susceptibility index (DSI) for final biomass and leaf area. A large number of traits were measured before and after the onset of a water deficit treatment, which were analyzed under several criteria: the significance of the regression with the DSI, phenotyping cost, earliness, and repeatability. The most efficient trait was found to be transpiration efficiency measured at 13 days after emergence. This trait was further tested in a second experiment with different water deficit intensities, and validated using a different set of genotypes against field data from a trial network in a third experiment. The framework applied in this work for assessing traits under different criteria could be helpful for selecting those most efficient for automated phenotyping.

## Introduction

Phenotyping is currently the bottleneck in breeding for many traits, including drought tolerance (Richards et al., 2010; Montes et al., 2011), mostly due to the cost of genotyping having largely decreased during the last years in relation to that of phenotyping. Conventional procedures for phenotyping complex traits are usually labor intensive, time consuming, low throughput, costly, and destructive to plants (Montes et al., 2007; Chen et al., 2014). High-throughput and reproducible phenotyping is then crucial for accelerating the release of improved varieties (Vadez et al., 2012). To help attain this goal, automated phenotyping platforms have been developed, which aim at increasing the capacity for obtaining phenotypic information. Greenhouse located plant phenotyping platforms are becoming increasingly widespread, due to the availability of commercial (e.g., Lemnatec, http://www.lemnatec.com; Photon Systems Instruments, http://www.psi.cz) and also commercial phenotyping services (e.g., the Plant Accelerator, https://www.plantphenomics.org.au/services/accelerator/). Efforts have been made toward lowering the initial cost of phenotyping platforms by developing affordable technologies (e.g., Pereyra-Irujo et al., 2012; Minervini et al., 2017). Operational costs of commercial and custom-made phenotyping platforms, however, have not received much attention, but can be crucial for assessing the practical applicability of this technology.

Besides costs, the relative value of the phenotypic data obtained should be considered, in comparison with traditional phenotyping techniques. Reproducible and precise phenotyping of early traits at medium or high throughput is a clear advantage of greenhouse located platforms. The main goal of phenotyping in a crop breeding context is usually to predict field performance, but doing so using automated platforms is usually questioned, mainly because environmental conditions in greenhouses and soil conditions in pots are often unrealistic (Poorter et al., 2012). Results on traits determined at vegetative stages on plants grown in pots in controlled environment experiments are difficult to relate directly into yield performance under field conditions (Junker et al., 2015), because in these conditions plants are far removed from the situation they will experience in the field (Araus and Cairns, 2014). Nevertheless, there are a few reports in the literature where phenotypic measurements in the greenhouse and the field are correlated. Chapuis et al. (2012) found a high genetic correlation between the sensitivity of grain number (the main component driving yield) to water deficit determined in a network of maize field trials and the sensitivity of leaf elongation rate in a greenhouse phenotyping platform. In soybean, Pardo et al. (2015) found similar rankings of water deficit tolerance of yield in greenhouse experiments and field trials and, though they did not use an automated phenotyping platform, their protocols and measurements could be readily automated. Another important aspect to take into account in order to estimate the value of phenotyping in a breeding context is the ability of a given trait to identify differences between genotypes, which can be quantified as the heritability (Specht et al., 2001; Rebetzke et al., 2002; Du et al., 2009) or repeatability, a concept better suited to cultivars from diverse sources and different pedigrees (Fehr, 1987; Lambrides et al., 2004; Hallauer et al., 2010). Also earliness (time between seedling emergence and trait measurement) could be useful when phenotyping cost is similar between two traits.

Obtaining plant genotypes with improved tolerance to drought is currently a main goal in plant breeding as water is the main factor limiting crop yield worldwide (Hufstetler et al., 2007). Conventional and marker-assisted breeding for improved tolerance to water deficit has been successful in different crops (e.g., maize, rice, wheat), through phenotyping in water-limited environments (Hall and Richards, 2013). However, this approach can be ineffective in changing environmental scenarios (i.e., climate change, Brisson et al., 2010). In addition, the time required for the development of an improved variety is long and very costly in resources (Hall and Richards, 2013). Therefore, accelerating breeding of drought tolerant varieties could bring enormous advantages, and efficient phenotyping is a key point to reach this goal. Most of the available phenotyping platforms automatically manipulate the soil water content through precise irrigation of each pot; therefore, it allows to measure the response to specific water deficit scenarios. They also provide measurements of a few basic traits frequently used for quantifying tolerance to water deficit, including growth traits estimated through the analysis of digital images of the plants (leaf area, aerial biomass, height), and water consumption. These data allow the calculation of indices useful for characterizing the responses of plants to drought, e.g., transpiration rate per unit leaf area and transpiration efficiency, leaf area ratio and net assimilation rate.

The objective of this work was to establish a framework for assessing the efficiency of phenotyping (i.e., the ratio of phenotyping value and cost) and applying it for selecting traits measured in an automated phenotyping platform aimed at predicting drought tolerance of field-grown soybean genotypes. The drought tolerance ranking of a set of soybean genotypes was first quantified in the GlyPh phenotyping platform, and compared to that previously determined under greenhouse and field conditions by Pardo et al. (2015). Second, different traits putatively associated with drought tolerance during the vegetative period were analyzed by estimating their phenotyping efficiency under different criteria. One of these traits was selected and further studied under different water deficit intensities. Finally, it was validated using a set of independent genotypes against field data obtained from a trial network and characterized under a specific protocol of analysis, by comparing data obtained in GlyPh against field data obtained from a trial network.

## Materials and methods

### Genetic material

Three experiments were carried out using different sets of soybean genotypes (Table 1). Experiment 1 included seven genotypes: five commercial genotypes (N7001, Munasqa, A8000, BR16, and TJ2049), one breeding line (XI73535RG), and one plant introduction (PI416937). All genotypes except XI73535RG had been previously evaluated by Pardo et al. (2015). Experiment 2 was carried out only with Munasqa and TJ2049, which were selected from results obtained in Experiment 1 and Pardo et al. (2015). Experiment 3 was carried out with a set of seven commercial genotypes: Bio 6.50, NS4611, RA644, SRM4222, SRM5200, SRM6001, and SRM6900, which were selected taking into account (i) covering a wide range of tolerance to water deficit in field conditions (ii) the availability of seeds (iii) the restrictions of size of the Glyph platform. Munasqa and TJ2049 were also included in this experiment as checks.

**Table 1:** Soybean genotypes used in the three experiments carried out in the GlyPh phenotyping platform.

**Genotype**	**Maturity group**	**Origin**	**Year of release**	**Type of germplasm**	**Growth habit**	**Experiment**
A8000RG	VIII	Argentina	1998	Cultivar	Determinate	1
BR16	VII	Brazil	1991	Cultivar	Determinate	1
Munasqa	VIII	Argentina	2001	Cultivar	Determinate	1, 2, 3
N7001	VII	USA	2000	Cultivar	Determinate	1
PI416937	V	Japan	1977	Plant introduction	Determinate	1
TJ2049	IV	Argentina	2003	Cultivar	Indeterminate	1, 2, 3
XI73535RG	VII	Argentina	–	Breeding line	Determinate	1
Bio 6.50	VI	Argentina	2011	Cultivar	Indeterminate	3
NS 4611	IV	Argentina	2012	Cultivar	Indeterminate	3
RA 644	VI	Argentina	2012	Cultivar	Determinate	3
SRM 4222	IV	Argentina	2012	Cultivar	Indeterminate	3
SRM 5200	V	Argentina	2012	Cultivar	Indeterminate	3
SRM 6001	VI	Argentina	2012	Cultivar	Indeterminate	3
SRM 6900	VI	Argentina	2012	Cultivar	Indeterminate	3

### Culture methods and growth conditions

The experiments were carried out using GlyPh, an automated phenotyping platform developed for soybean which allows the evaluation of genotypes under precisely controlled water deficit conditions (described in detail in Pereyra-Irujo et al., 2012). GlyPh allows the evaluation of up to 120 plants growing in individual pots; automatic watering and measurement routines allow the simulation of multiple water regimes for each plant individually, and the measurement of soil water content and image capture for growth estimation. Plants were initially grown in a growth chamber [16 h photoperiod, 300–600 μmol m^−2^ s^−1^ PAR, 24/19°C temperature day/night, 1.9/1.18 KPa vapor pressure deficit (VPD) day/night]. Four days after emergence (DAE), plants were transferred to GlyPh. The platform is located at the Balcarce Experimental Unit of the National Institute of Agricultural Technology and the Faculty of Agricultural Sciences (UNMdP) in an environmentally controlled glasshouse (S37°46′, W58°18′), where heaters and coolers were set to start at 13°C and at 27°C, respectively. Plants were grown in cylindrical PVC pots (10 cm diameter, 35 cm high) filled with soil. This soil was an A horizon from a Typic Argiudoll soil. Each pot was sown with two seeds, and at the V1 stage (Fehr and Caviness, 1977) seedlings were thinned to one plant per pot. Inoculation with *Bradyrhizobium japonicum* was not carried out to avoid possibly confounding effects from biological nitrogen fixation or from its response to water deficit (Serraj et al., 2001). Nutrient solution (Hoagland solution at 100%) was applied by irrigation every 4 days. 100% Hoagland's solution contains 15 mol m^−3^ of NO_3_, a concentration higher than that required for inhibiting nodulation (4 mol m^−3^ of NO_3−_; Harper and Gibson, 1984; Imsande, 1986). Nodules in the root system were not observed along the experiment. Initial soil water content was measured by oven-drying samples at 105°C for 48 h and subsequently controlled by automated weighing, as described in Pereyra-Irujo et al. (2012). Pots were initially watered to a mean soil water content of 0.26 g water g soil^−1^ which corresponds to a soil water potential of −0.033 Mpa (the soil moisture retention curve was previously characterized in the laboratory; INGEIS, CONICET-UBA, Buenos Aires, Argentina), and maintained through daily irrigations. Daily changes in pot weight were attributed to changes in soil water status after correction for plant weight; for this correction, two plants per treatment were harvested and weighed weekly as described by Granier et al. (2006). Photosynthetically active radiation, relative humidity, and air temperature were measured every 15 min, and averaged every 1 h, with dataloggers (Four Channel Datalogger, Cavadevices, Buenos Aires, Argentina). Vapor pressure deficit was calculated from temperature and relative humidity data. Mean values of meteorological conditions during each experiment are presented in Table 2 (Supplementary Figure 1).

**Table 2:** Meteorological conditions during the three experiments in the phenotyping platform GlyPh.

**Experiment**	**Day length (h)**	**PAR (μmol m^−2^ s^−1^)**	**T day/night (°C)**	**RH day/night (%)**	**VPD day/night (KPa)**
1	14	387	28/17	57/85	1.63/0.31
2	14	292	21/12	43/56	1.42/0.70
3	14	239	24/17	46/59	1.74/0.78

*Mean values of day length, incident solar radiation (PAR), temperature (T), relative humidity (RH), and vapor pressure deficit (VPD), averaged for the whole experiment period*.

In Experiment 1, between 12 and 17 plants of each genotype were grown. All plants were initially grown under well-watered conditions (WW, 0.26 g water g soil^−1^, −0.033 MPa), and at 33 DAE, a water deficit treatment (WD, 0.21 g water g soil^−1^, −0.21 MPa) was randomly imposed to half of the pots. Irrigation of pots was stopped until the desired soil water content was reached. Plants were harvested at 57 DAE.

In Experiment 2, 16 plants each of Munasqa and TJ2049, the two genotypes with most contrasting behavior under WD were grown. Plants were initially maintained under well-watered conditions (0.26 g water g soil^−1^, −0.033 MPa). At 34 DAE, four randomly-chosen plants of each genotype were subjected to each of four soil water content treatments: 0.26 g water g soil^−1^ (−0.033 MPa, WW), 0.21 g water g soil^−1^ (−0.21 MPa, WD1), 0.19 g water g soil^−1^ (−0.65 MPa, WD2), and 0.18 g water g soil^−1^ (−0.94 MPa, WD3). The soil water content corresponding to each water deficit treatment was reached at different moments (34, 43, and 44 DAE for WD1, WD2, and WD3, respectively). Although both genotypes reached the target water deficit at different dates for the different water treatments (average differences = 2.67 ± 1.3), this differences were not significant (*p* = 0.32, 018, and 0.35 for WD1, WD2, and WD3, respectively). Plants were harvested at 57 DAE.

In Experiment 3, between 8 and 12 plants of seven commercial genotypes were grown (Table 1). Plants were grown under well-watered conditions (0.26 g water g soil^−1^, −0.033 MPa) and harvested at 27 DAE. In this experiment the WD treatment was not included because the traits to be validated corresponded to well-watered conditions.

### Measurement of traits

Several phenotypic traits were evaluated during the vegetative stage with GlyPh (Table 3). These traits were defined by the multiple combinations of the basic trait measured, the measurement time, and the soil water content treatment.

**Table 3:** Phenotypic traits measured in Experiment 1.

**Category**	**Trait**	**Measurement time (DAE)**
Morphology	Leaf dry weight	57
	Stem dry weight	57
	Leaf dry weight of branches	57
	Stem dry weight of branches	57
	Leaf area	13, 20, 27, 33, 38, 44, 57
	Shoot dry weight	13, 20, 27, 33, 38, 44, 57
	Number of nodes	57
	Number of branches	57
	Leaf area ratio (LAR)	33, 38, 44, 57
	Specific leaf area (SLA)	57
Biomass partitioning	Leaf mass ratio (LMR)	57
	Stem mass ratio (SMR)	57
	Leaf mass ratio of branches (LMR_b_)	57
	Stem mass ratio of branches (SMR_b_)	57
Growth	Relative expansion rate (RER)	33, 38, 44, 57
	Relative growth rate during WS (RGR_WS_)	57
	Net assimilation rate (NAR)	57
Water use	Total transpired water	57
	Transpiration (T)	Daily
	Transpired water during WS	57
	Transpiration efficiency (TE)	13, 20, 27, 33, 38, 44, 57
	Transpiration rate at break Point	54
	Transpiration rate at max VPD	54
	Transpiration rate (TR)	50, 51, 52, 53, 54
	Slope1^*^	54
	Slope2^*^	54
	Slope2:Slope1^*^	54
	Intercept 1^*^	54
	Intercept 2^*^	54
	Leaf-to-Air temperature difference	17, 18, 24, 37, 41, 42, 43, 46, 49, 50, 52, 53, 57
	Leaf temperature	17, 18, 24, 37, 41, 42, 43, 46, 49, 50, 52, 53, 57
	Stomatal conductance (g*s*)	17, 24, 37, 46

* *Slopes and intercepts of a two-segment linear regression representing the response of TR to VPD*.

In Experiment 1, leaf area (LA) was estimated by manually measuring the width and length of all terminal leaflets, as in Wiersma and Bailey (1975), at 13, 20, 27, 33, 38, and 44 DAE. Shoot dry weight (SDW) was estimated as a function of leaf area and plant age, the calibrated function used was as described in Pereyra-Irujo et al. (2012):

(1) $$\begin{array}{l} \text{SDW}(\text{g})=0.1+0.00184^{*}\text{LA}({\text{cm}}^{2})+0.0000926^{*}\text{LA}({\text{cm}}^{2})\\ \;*\text{Plant age}(\text{days}) \end{array}$$

In Experiments 2 and 3, LA was non-destructively estimated from image data, obtained from automated imaging routines carried out with GlyPh. Top and side view images were obtained for each plant at 13, 27, 33, 38, 44, and 49 DAE in Experiment 2, and at 13, 20, and 27 DAE in Experiment 3. Images were segmented and the number of pixels corresponding to plant material were counted using the ImageJ image analysis software (Abramoff et al., 2004). Data were converted to cm^2^ using the corresponding calibration factor for each camera and summed to obtain the projected shoot area as in Pereyra-Irujo et al. (2012). Projected shoot area was highly correlated to manually measured leaf area (*R*^2^ = 0.98, RMSE = 15.6 cm^2^, data not shown). Shoot dry weight was estimated by using Equation (1).

At the end of Experiments 1 and 2 (57 DAE), plants were harvested, and separated into main stem and branches. Dry weight of these shoot fractions was determined after drying at 50°C to constant weight. Branches and nodes were counted. Allometric ratios were calculated by dividing the mass of leaves and stems (either for the whole plant or only the branches) by final shoot dry mass (LMR, SMR, LMRb, and SMRb, respectively).

Total transpired water was calculated as the sum of daily evapotranspiration minus soil evaporation determined through the automated daily weighing of the pots in GlyPh. Three pots without plants, filled with the same soil, were placed in the platform to measure direct water evaporation from the soil. Transpiration efficiency (TE) was estimated as the ratio between shoot dry weight and the total transpired water accumulated from 0 DAE. TE was determined at 13, 20, 27, 33, 38, 44, and 57 DAE in Experiment 1, at 13, 27, 33, 38, 44, and 57 DAE in Experiment 2 and at 13, 20, and 27 DAE in Experiment 3.

In Experiment 1, other water use, morphology and growth traits were also measured. Specific leaf area (SLA) was calculated as the ratio between plant leaf area and dry mass of leaves; Leaf area ratio (LAR) was calculated as the ratio between leaf area and the shoot dry weight.

Relative expansion rate (RER) was calculated as:

$$\begin{array}{l} RER=\left(\frac{\ln\;LA_{2}-\ln\;LA_{1}}{t_{2}-t_{1}}\right) \end{array}$$

Where *LA*, leaf area; *t*, time; subscripts 1 and 2 correspond to successive collection. Net assimilation rate (NAR) was calculated from the following expression:

$$\begin{array}{l} NAR=\left(\frac{SDW_{2}-SDW_{1}}{t_{2}-t_{1}}\right)÷\left(\frac{\ln\;LA_{2}-\ln\;LA_{1}}{LA_{2}-LA_{1}}\right) \end{array}$$

Where *SDW*, shoot dry weight, *LA*, leaf area; *t*, time; subscripts 1 and 2 correspond to successive collection.

The response of transpiration rate (TR, mg water m^−2^ s^−1^) to air vapor pressure deficit (VPD) was measured. During 5 consecutive days, plants in both well-watered and water deficit treatments were sequentially weighed at regular time intervals between 11:00 and 14:00 h, with VPD ranging between 1.2 and 2.98 KPa. Transpiration rate for each genotype was regressed against VPD. A two-segment linear regression (Fletcher et al., 2007) was applied to the data using GraphPad Prism 5 (GraphPad Software Inc., San Diego, CA)^1^ for each genotype:

$$\begin{array}{l} \text{If VPD}<\text{BP},\text{TR }={\text{I}}_{1}+{\text{S}}_{1}\left(\text{VPD}\right) \end{array}$$

$$\begin{array}{l} \text{If VPD }\geq \text{ BP},\text{TR }={\text{I}}_{2}+{\text{S}}_{2}\left(\text{VPD}\right) \end{array}$$

where BP is the breakpoint between the two linear segments. A common BP was established for all genotypes (2.47 ± 0.14 KPa), as the average BP from previously adjusted two-segment linear regression for each genotype. Parameters of two segment linear regressions were estimated for each plant of each genotype: intercept 1 (I_1_), intercept 2 (I_2_), slope 1 (S_1_), slope 2 (S_2_), and the change in slope at high VPD (S_2_:S_1_ ratio). In addition, the TR at maximum VPD (TR_maxVPD_) and TR at the breaking point (TR_BP_) were calculated for each plant of each genotype.

Stomatal conductance (g*s*) was measured using a porometer (Decagon SC-1, Decagon Devices, Pullman, WA). Measurements were taken on the abaxial side of the youngest fully expanded leaf of the main stem. In Experiment 1 *g*_*s*_ was measured at 17, 24, 37, and 46 DAE. Leaf temperature was measured in all plants at 17, 18, 24, 37, 41–43, 46, 49, and 50–53 DAE using an infrared thermometer (Omega model OS-FS, Stamford, CT). Thermal measurements were performed at midday on the adaxial side of two fully expanded leaves from the top of the main stem, and averaged. The difference between leaf and air temperature was also calculated (Idso, 1982).

For all traits measured both under WW and WD conditions, the drought susceptibility index (DSI) was calculated as in Du et al. (2009).

$$\begin{array}{l} DSI=\left(1-\frac{Ywd}{Yww}\right)/\left(1-\frac{Xwd}{Xww}\right) \end{array}$$

where *Y*_*wd*_ and *Y*_*ww*_ are trait values for a given genotype, and *X*_*wd*_ and *X*_*ww*_ are the means of all genotypes, under WD and WW conditions. In Experiments 1 and 2, the DSI for final shoot dry weight and leaf area was used as a measurement of (the inverse of) drought tolerance of each genotype for subsequent analyses.

### Evaluation of phenotyping efficiency and selection of traits

In order to identify those traits with the highest phenotyping efficiency from data obtained in Experiment 1, four criteria were considered:

- The regression between each trait and the target trait (the trait intended to be predicted, the DSI for final dry weight in this case), discarding those traits with non-significant relationships (*p* > 0.05).
- The ratio between the determination coefficient of the relationship between the trait and the target trait and the relative phenotyping costs (the ratio of the costs of the trait and the target trait), as in Medugorac and Soller (2001), discarding those with a ratio lower than 2 (assuming the preference of an indirect trait should be justified by being largely advantageous). Assuming the time and space allocation in a phenotyping platform to be the main cost, the phenotyping cost of each trait was approximated by calculating the product of the number of replicates used and the time required to reach the moment of that measurement (plant·day).
- Earliness (in days after emergence) was used as an additional selection criteria (independently of cost), since it implies an inherent advantage in phenotyping throughput. The earlier traits among those that fulfilled the previous criteria were selected.
- The ability to detect differences between genotypes assessed through the repeatability (w^2^) (Fehr, 1987), for each trait. The repeatability of a given trait was considered acceptable when w^2^ was similar or higher than 0.5.

Before onset of drought treatment, w^2^ was calculated as:

$$\begin{array}{l} {\text{w}}^{2}=\frac{\sigma_{g}^{2}}{\sigma_{g}^{2}{+}^{\sigma_{e}^{2}}{/}_{r}} \end{array}$$

where $\sigma_{\text{g}}^{2}$ is the genotypic variance $\sigma_{e}^{2}$ environmental variance and r is the number of replicated plants. After onset of drought treatment, w^2^ was calculated as:

(8) w2σg2σg2+ σe2rt+ σge2t

where $\sigma_{\text{ge}}^{2}$ is the genotype × environment variance, and t is the number of environments (i.e., soil water content treatments). Variance components were estimated by residual maximum likelihood (REML) procedure in SAS (SAS, 2017).

### Validation of the selected trait against field trial data of independent genotypes

A database was created from published data obtained from Argentina's national trial network of soybean cultivars (Red de Ensayos de Cultivares de Soja, RECSO, INTA, http://inta.gob.ar). This network extends from 23 to 38°S and from 58 to 65°W, covering a wide range of environmental conditions during the soybean growing season, and a wide range of soil types varying from sandy loam to clay loam (Panigatti, 2010). The database included data obtained from 80 environments (Supplementary Table 2), as the result of combining years (2012–2016) and locations, and a total of 282 cultivars. During the crop cycle, rainfall was between 212 and 1,178 mm and average daily temperature between 17 to 24°C. Input data were genotype, sowing, emergence and physiological maturity dates, monthly rainfall, and grain yield.

In order to evaluate the response of cultivars to water availability, environments where yield was mainly limited by water were identified, based on the relationship between grain yield and water input. For each environment, water input was quantified as the average total rainfall, from 60 days before sowing until physiological maturity and yield as the average yield of the genotypes sowed in this trial. The model proposed by Bouman and Toung (2001) was used:

$$\begin{array}{l} Yield=a\left(1-e^{\left(b\left(water\;input-c\right)\right)}\right) \end{array}$$

Where *a* is the attainable yield, *b* the initial factor-use efficiency, and *c* the theoretical minimum amount of the input factor needed for any yield at all. The model was fit iteratively so that 95% of the environments were below the curve. The environments with an average yield between −20 and +20% of this curve were considered as “water-limited,” while the rest of the environments were assumed to have been limited by other factors, and not considered in further analyses.

The tolerance to water deficit of each genotype was quantified using two approaches. First, selected environments were further classified as wet, intermediate and dry environments. The cut off points were chosen according to the amount of rainfall during the period between 3 months and 1 month before harvest; dry years were those with rainfall of <175 mm during the critical period, and wet years period were those with at least double the rainfall, as in Pardo et al. (2015). The DSI was calculated (using Equation 6) for each genotype considering its average yield in the dry and wet environments. The second approach included: (i) calculating, for each genotypes, the difference between its actual yield and the average yield of each environment (ΔY) was calculated, and (ii) regressing the ΔY values against water input; the slope of this relationship was used then as an estimate of tolerance to water deficit (genotypes with positive slope were considered sensitive, while those with negative slope were considered tolerant). Based on these data, seven genotypes were chosen considering (i) covering a wide range of tolerance to water deficit in field conditions, (ii) the availability of seeds, and (iii) the restrictions of size of the GlyPh platform. These genotypes were grown in Experiment 3, and the trait previously selected from in Experiments 1 and 2 was measured for its validation against field data.

### Data analysis

The data obtained from Experiments 1 and 2 were analyzed using the PROC MIXED procedure in SAS (SAS Institute Inc., Cary, NC). Water regimes and blocks were considered as fixed effects, and genotypes and replicates were considered as random effects. Data from Experiment 3 was analyzed by analysis of variance (ANOVA). Differences between genotypes means were analyzed with Tukey-Kramer test (*P* < 0.05). DSI for SDW and LA values from Experiment 1 were used to compare the tolerance to DSI from yield in greenhouse experiments and field trials reported by Pardo et al. (2015) (*p* < 0.05). In Experiment 3, the most efficient trait was validated by regression against DSI for yield and the slope of ΔY (*p* < 0.05).

## Results

### Drought susceptibility index for shoot dry weight during the vegetative period correlated with published DSI for yield

**Figure 1:**
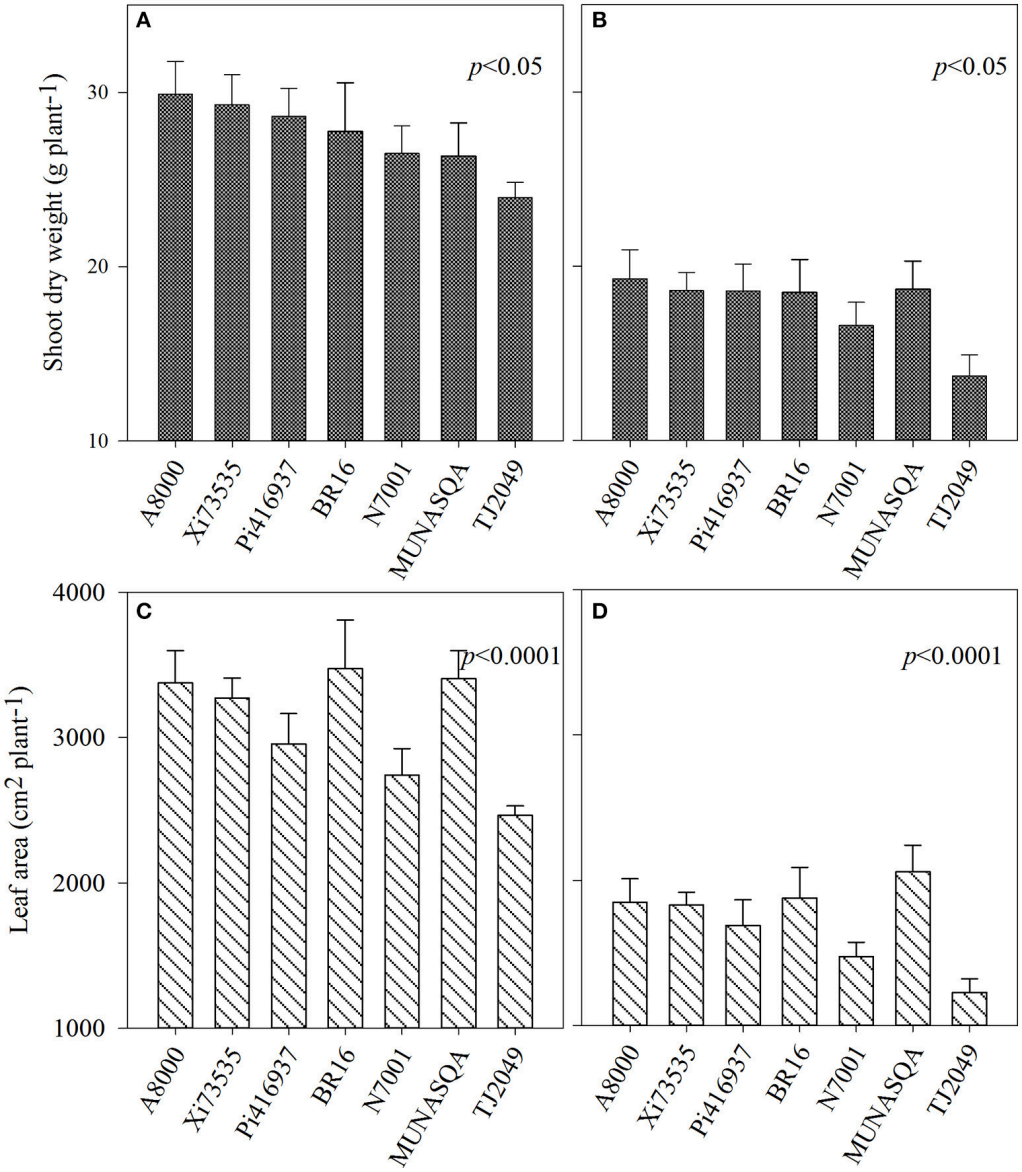
Shoot dry weight **(A,B)** and leaf area **(C,D)** at 57 days after emergence of the Experiment 1 for the seven soybean genotypes under well-watered (left) and water deficit conditions (right). Vertical bars represent mean values, error bars represent standard errors.

Shoot dry weight and leaf area at 57 DAE differed significantly between genotypes in Experiment 1 (*p* < 0.05 and *p* < 0.0001, respectively, Figure 1). Under water deficit, SDW decreased between 29 and 43% (for Munasqa and Tj2049, respectively) and LA between 39 and 50% (Munasqa and Tj2049, respectively).

**Figure 2:**
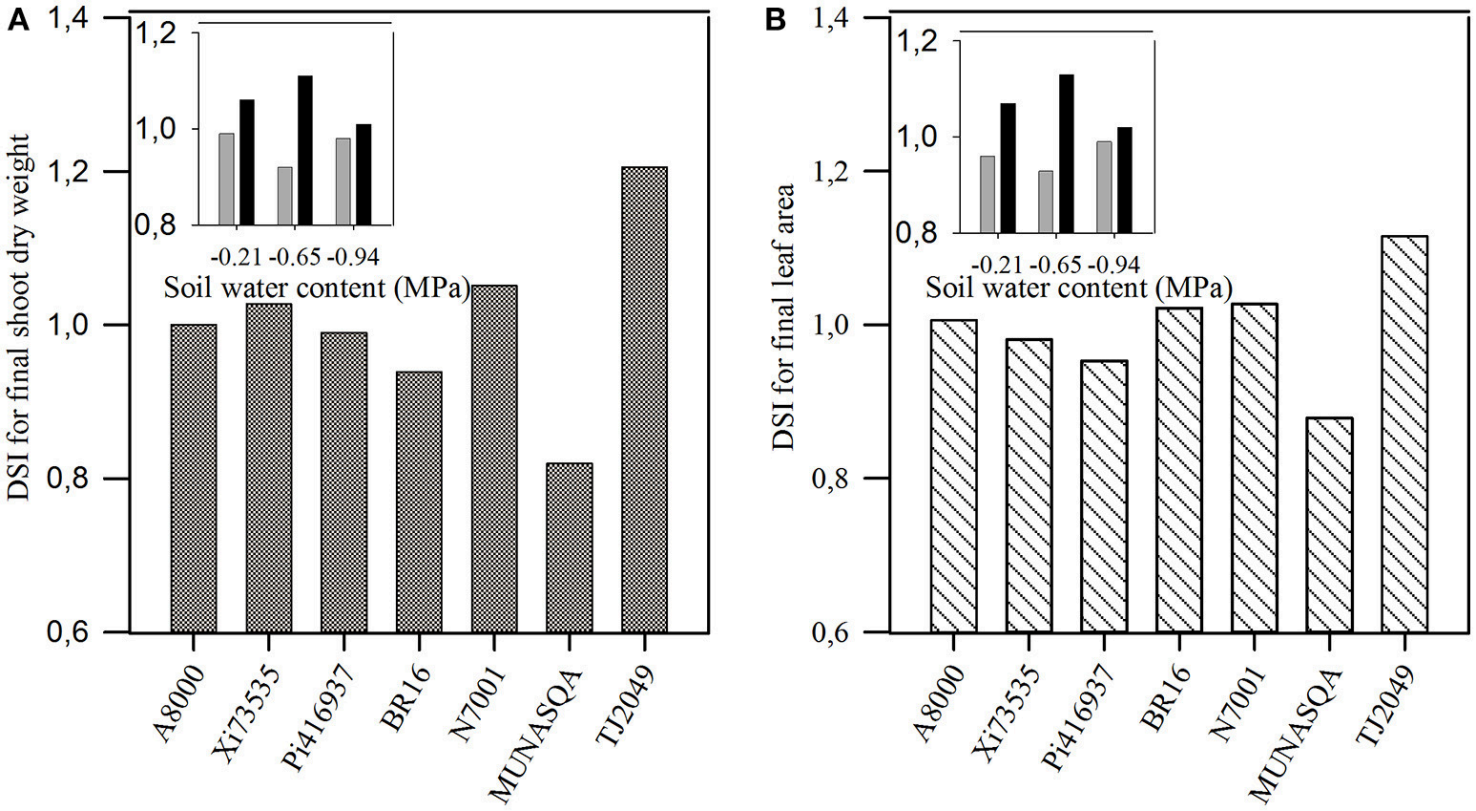
Drought susceptibility index (DSI) for: **(A)** shoot dry weight and **(B)** leaf area at 57 days after emergence (DAE) in Experiment 1. Insets: DSI for shoot dry weight at 57 DAE for contrasting genotypes (Tj2049, black bars and Munasqa, gray bars) under three soil water deficits (−0.21, −0.65, and −0.94 MPa) in Experiment 2.

The DSI for both traits, calculated for Experiments 1 and 2 are presented in Figure 2. The DSI ranged from 0.82 to 1.21 in Experiment 1 and from 0.92 to 1.13 in Experiment 2. The DSI for final SDW and SDW at 57 DAE under WW were not associated with SDW at 57 DAE under WW conditions (*p* = 0.39), while a negative association with SDW at 57 DAE under WD condition was found (*R*^2^ = 0.63, *p* = 0.03).

The DSI for final SDW correlated with yield DSIs found by Pardo et al. (2015) in greenhouse conditions (Experiments 2 and 3 from Pardo et al., 2015; *r* = 0.81, *p* < 0.05 and *r* = 0.97, *p* < 0.05, respectively) and in field trials (*r* = 0.99, *p* < 0.10). Similar results were found using the DSI for final LA. In Experiment 1, TJ2049 showed the highest DSI for both SDW and LA, while Munasqa had the lowest index for both traits (Figures 2A,B). These genotypes showed the same behavior when their DSI for SDW and LA were tested across different levels of water deficit (insets in Figures 2A,B). Based on these results, these two contrasting genotypes were further analyzed in Experiment 2.

### Selection of the most efficient trait

**Figure 3:**
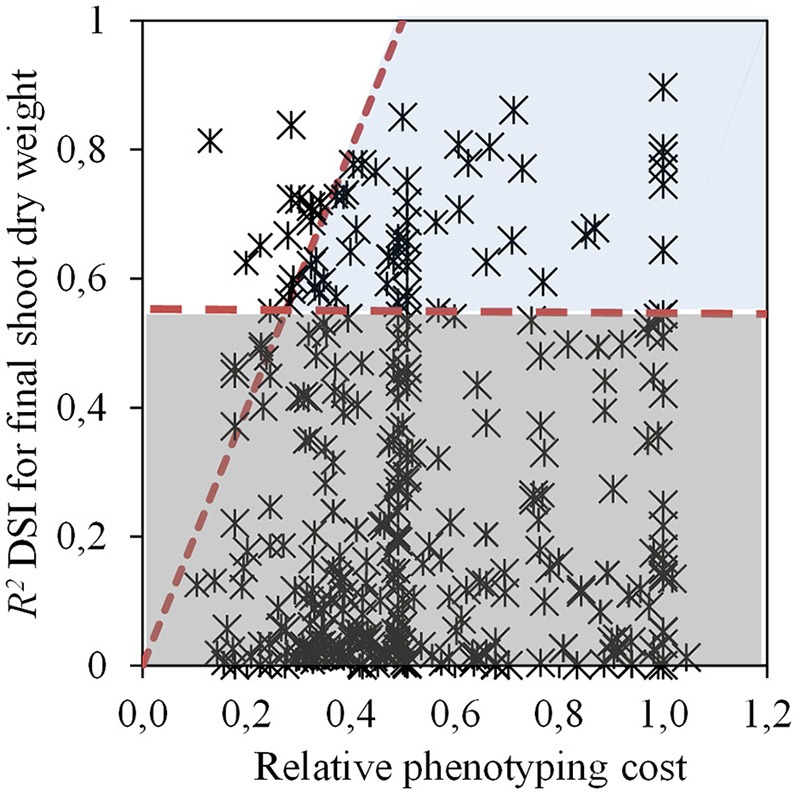
Determination coefficient (*R*^2^) of the relationship between drought susceptibility index (DSI) for shoot dry weight at 57 DAE and a given trait vs. relative phenotyping cost (calculated as the phenotyping cost of each trait divided by the cost of DSI for shoot dry weight). Horizontal dash line indicates the threshold for significant regressions. Diagonal dash line indicates the threshold of the ratio of the determination coefficient of the relationship of DSI for shoot dry weight to the relative phenotyping cost. White section shows the significant and selectable relationships (*p* < 0.05), the dark gray section of the figure exhibit the non-significant relationships (*p* > 0.05) while the light gray area displays the traits discarded for having a ratio between the determination coefficient and the relative phenotyping cost, lower than 2. Data from Experiment 1.

In Experiment 1, several traits were evaluated with GlyPh during the vegetative stage, from 13 to 57 DAE. Four different selection criteria were used in order to select those traits with the highest phenotyping efficiency. The first criterion was the significance of regressions between each evaluated trait and the DSI for final biomass (the target trait in this case), leading to the selection of 64 trait per moment per treatment combinations (from a total of 358, Figure 3, Supplementary Table 1). These traits were subsequently evaluated with the second criterion, the ratio between the determination coefficient of the relationship between each trait and the target trait, and the relative phenotyping cost. A total of 13 trait per moment per treatment combinations presented a ratio ≥2 (Figure 3), with traits corresponding to the morphology (LA, SDW, and LAR) and water use (T, TE, TR, and gs).

**Figure 4:**
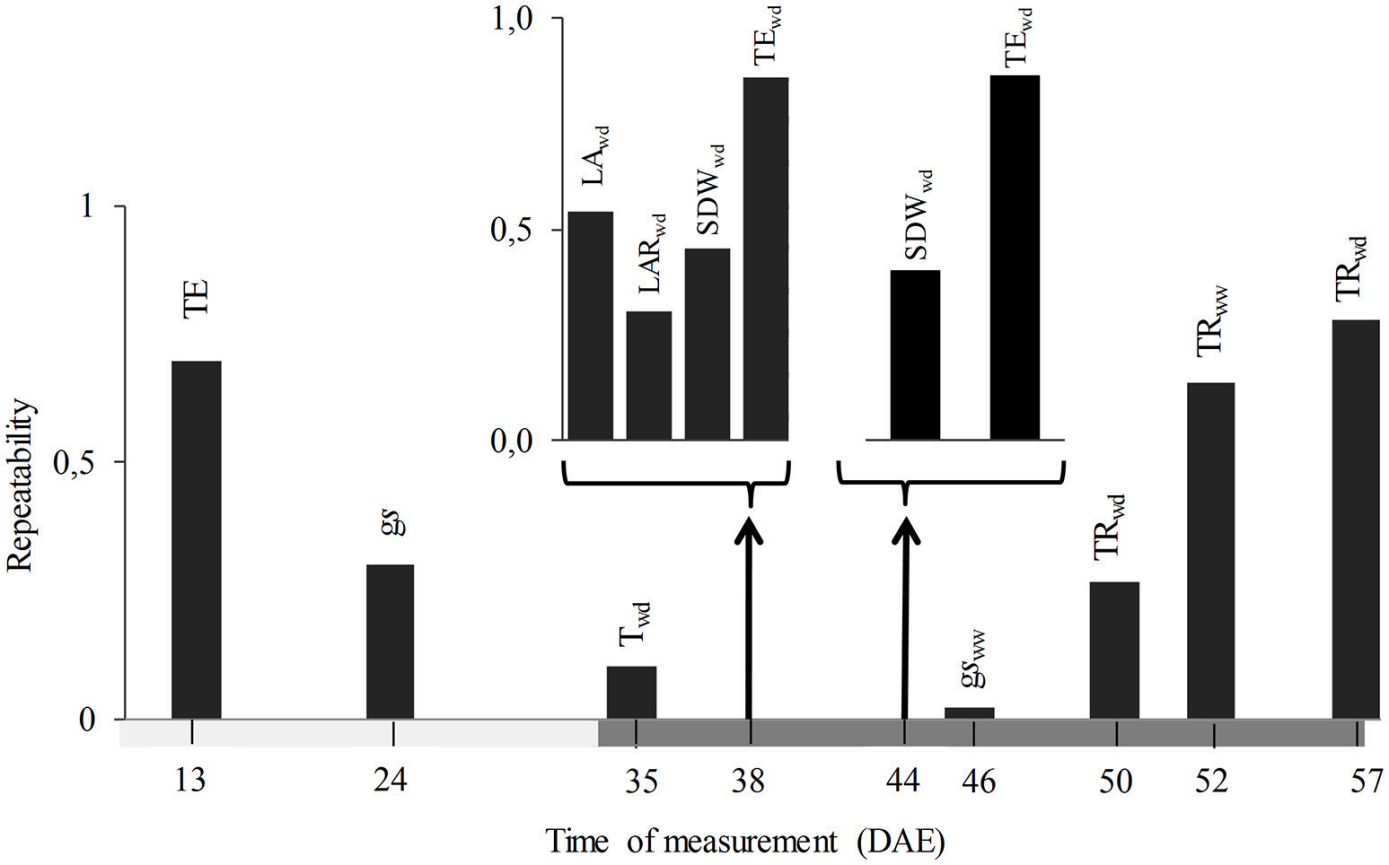
Repeatability (upper bar graphs) and time of measurement (DAE: days after emergence, down timeline) of the traits selected by the ability to predict drought tolerance of a given genotype. Horizontal dark gray bar indicates the length of the water deficit treatment. TE, transpiration efficiency; g*s*, stomatal conductance; T, transpiration; SDW, shoot dry weight; LAR, leaf area ratio; TR, transpiration rate; WW and WD, well watered and water deficit treatments respectively.

The third selection criterion used was earliness, finding that measurement time of the traits so far selected ranged between 13 and 57 DAE, with two of them measured before imposition of water treatments (TE at 13 DAE and g*s* at 24 DAE). When evaluated according to the last selection criterion, it was found that g*s* presented a low repeatability (0.30, Figure 4), while that of TE was higher, not only at 13 DAE (0.70), but also at 38 and 44 DAE (0.86 for both). According to results of applying the four criteria, TE at 13 DAE was selected as the most efficient trait and subjected to further analysis and validation against field data using independent genotypes.

### Characterization of the selected trait

**Figure 5:**
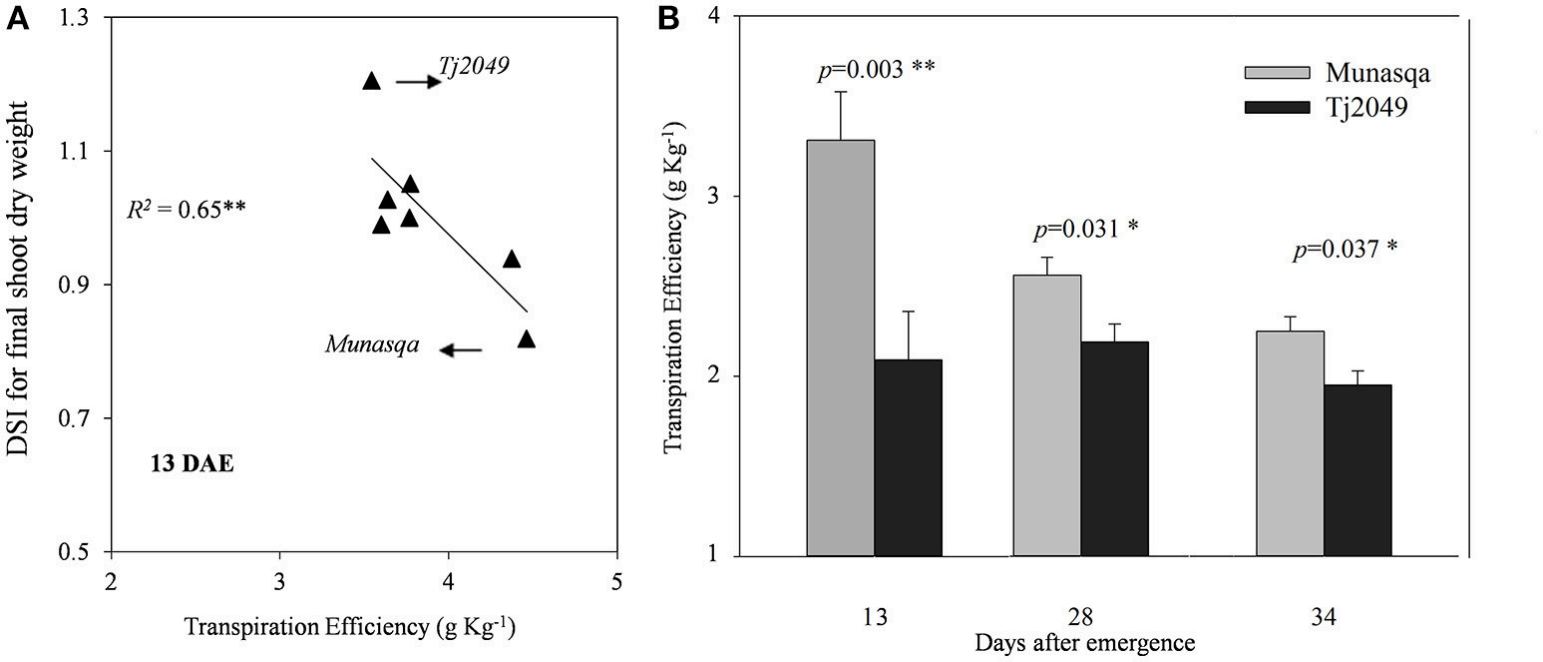
**(A)** Relationship between Drought Susceptibility Index (DSI) for final shoot dry weight and transpiration efficiency at 13 days after emergence (DAE) under well watered conditions in the Experiment 1.**(B)** Transpiration efficiency for Munasqa (gray columns) and Tj2049 (black columns) contrasting genotypes in the well-watered treatment at 13, 27, and 33 DAE in the Experiment 2. Bars represent mean values, error bars represent standard errors. Significant differences (*p* < 0.05 and *p* < 0.01) are represented as * and **, respectively.

In Experiment 1, TE at 13 DAE showed an inverse relationship with the DSI for final SDW (Figure 5A). The drought-tolerant genotype (Munasqa) showed the lowest DSI value and the highest TE, while the drought-susceptible genotype (TJ2049) showed opposite values. In Experiment 2, the drought-tolerant genotype Munasqa displayed significantly higher TE than the drought-sensitive genotype, TJ2049 at 13, 27, and 33 DAE in plants under WW conditions (Figure 5B).

### Validation of the selected trait against data from field trials for independent genotypes

**Figure 6:**
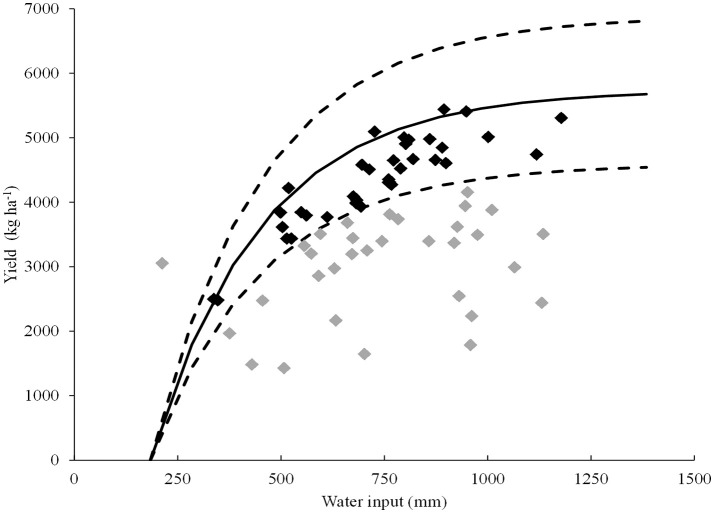
Relationship between average grain yield and water input during the whole crop cycle for 80 environments evaluated from the Argentina's national trial network of soybean cultivars (RECSO) database. The curve with solid line represents adjusted yield (Equation 9) and curves of dotted lines represents ±20% of the potential yield. Each data point represents one of the 80 environments. Black data points represent water limited environments while gray data points represent environments with limitations to yield besides water (these environments where excluded from the study).

To validate the ability of the trait TE at 13 DAE for predicting the tolerance of water deficit in independent genotypes, a group of seven genotypes was selected based on field yield data. A relationship between grain yield and water input for 80 field environments was established, and 36 water-limited environments were selected (Figure 6). The selected environments were in the range of 24 to 38°S and 58 to 64°W. Water input during the whole cycle was in the range of 338–1,178 mm and the average yields per environment varied between 2,478 and 5,440 kg ha^−1^. According to Pardo et al. (2015), these environments were then classified as dry (*n* = 14), intermediate (*n* = 16), and wet (*n* = 6). Mean yield in the dry environments ranged between 3,387 and 3,980 Kg ha^−1^ for the seven genotypes evaluated, whereas the average yield under wet conditions was between 4,049 and 5,103 Kg ha^−1^ (Supplementary Figure 2). The DSI calculated for each genotype considering its average yield in the dry and wet environments ranged from −0.70 to 2.48, and the slope of ΔY (see section Validation of the Selected Trait Against Field Trial Data of Independent Genotypes) ranged from −6.67 to 3.17. A negative association between DSI for yield and mean yield in the dry environments was found (*r* = −0.80, *p* < 0.0001), while a positive correlation was found with mean yield in the wet environments (*r* = 0.30, *p* = 0.00004), meaning that DSI for yield is mainly explained by the yield under drought.

**Figure 7:**
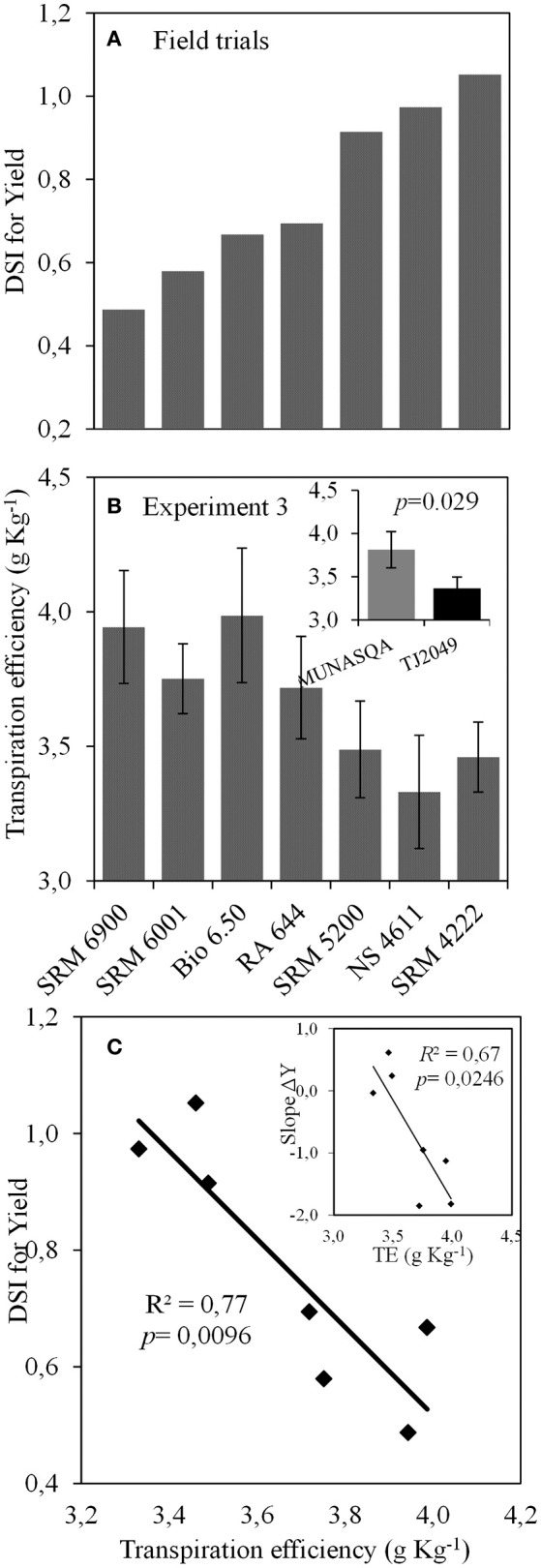
**(A)** Values of drought susceptibility index (DSI) for yield (data from RECSO database), **(B)** transpiration efficiency at 13 days after emergence (DAE) for the seven genotypes. Inset: Transpiration efficiency for Munasqa and Tj2049 contrasting genotypes. Bars represent mean values; error bars represent the standard error. Data from Experiment 3. **(C)** Relationship between DSI for yield and transpiration efficiency at 13 DAE. Inset: relationship between Slope of ΔY and transpiration efficiency at 13 DAE. ΔY is the difference between actual yield and the average yield of each environment, TE is the transpiration efficiency at 13 DAE.

Seven genotypes were selected based on this data. DSI values for yield ranged from 0.49 to 1.05, being minimum for SRM6900 and maximum for SRM4222, and values for the slope of ΔY ranged from −1.9 to 0.6 (Figure 7A). The value of TE at 13 DAE was higher for Munasqa than for TJ2049, the contrasting genotypes identified in Experiments 1 and 2 (inset in Figure 7B). For the seven genotypes selected from the field trial network, TE at 13 DAE ranged from 3.46 to 3.99 g Kg^−1^ (Figure 7B), and was negatively correlated with the DSI for yield (*R*^2^ = 0.77, Figure 7C) and with the slope of ΔY (*R*^2^ = 0.67, inset in Figure 7C). Furthermore, the DSI for yield and the slope of ΔY were also significantly correlated with TE at 21 (*R*^2^ = 0.68 and 0.70, respectively) and 28 DAE (*R*^2^ = 0.69 and 0.70, respectively, data not shown).

## Discussion

Given that greenhouse phenotyping aimed at predicting field performance is usually questioned, in this work we performed two different validations. First, the ranking of the most contrasting genotypes from previously published field data was found to be conserved under different levels of water deficits applied using GlyPh. While Pardo et al. (2015) compared the sensitivity of yield to drought between greenhouse and field conditions, our results show that a trait measured in the vegetative stage could also be valid for predicting the DSI of yield. Since this analysis was based on a limited number of genotypes, and the validity of the results could be restricted by the variability in this sample, the selected trait was validated against independent field data and a different set of genotypes. To the best of our knowledge, only the work of Chapuis et al. (2012) in corn had previously found a correlation between the sensitivity to water deficit of grain number determined in a network of field trials and the sensitivity of a vegetative trait measured in a phenotyping platform. Other potentially valuable traits could have been also validated, especially those included in Figure 4. These traits were either related to the amount of leaf area, aerial biomass, and the ratio between them (LAR) under water deficit, and the transpiration rate in the late stages of the experiment. The stomatal conductance was also an interesting candidate trait, but showed a lower repeatability. TE at 13 DAE was chosen as an ideal trait for validation, being easy to measure and likely encompassing the effects of biomass accumulation and stomatal behavior, but further studies need not be limited to only one trait.

Field trial networks are carried out in many countries, and they provide simple and accessible data. This kind of data source could avoid the need of experimental work to obtain data for validation in phenomic studies. In this work we propose a technique to identify those environments which are limited mainly by the amount of available water, based on the equation proposed by Bouman and Toung (2001), but which could be similarly applied to other factors for which data were available (e.g., nutrients). The data analysis was simple involving the use of the envelope curve (de Wit, 1992), the critical period for yield (Fischer, 1975; Jiang and Egli, 1993), a rule of thumb for identifying dry and wet environments (Pardo et al., 2015), and the susceptibility index DSI to quantifying water deficit tolerance (Du et al., 2009). Two different methods were used to calculate the sensitivity of each genotype to water availability: one based on the slope of relative yields against water availability, and the other based on a DSI calculated using contrasting environments. Both yielded similar results, but the latter showed a slightly higher correlation to greenhouse data. The applied procedure allowed identifying genotypes with a wide range of tolerance to water deficit. The data set for validating TE at 13 DAE was completed by measuring the trait in GlyPh in Experiment 3, automatically determining the shoot dry weight by imaging and total transpired water by weighing. The applied procedure was successful for validating the selected trait. Thus, the use of widely available data sources and combining them with classical approaches of data analysis in agronomy, ecophysiology and plant breeding with precise measurement of traits available in most of greenhouse automated phenotyping platforms could be useful to answers questions not currently unraveled in phenomics.

The selected trait, TE at 13 DAE, was found to be correlated with field data despite the wide variation of environmental conditions in the trial network (e.g., temperature, soil type), suggesting a low effect of the interaction between water availability and other environmental factors on water deficit tolerance in the studied genotypes. This trait correlated with the response of yield to water availability, even though it was measured early in the crop cycle and under well-watered conditions, in agreement with Earl (2002) and Pereyra-Irujo et al. (2012) which found TE in soybean to be a constitutive trait.

In the work of Chapuis et al. (2012) in corn, the most probable mechanism accounting for the correlation between results obtained in a phenotyping platform and in the field was that leaf growth, silk growth and the anthesis–silking interval share part of their genetic determinism (Welcker et al., 2007). Our results suggest that high TE could be a mechanism that could be underlying drought tolerance under the field conditions explored. Tardieu (2012) considers that high TE is only useful to tolerate severe terminal water stress. In the database used, the “dry” environments received in average 607 ± 53 mm, enough to yield near 3,864 kg ha^−1^, with only 13% of environments with rainfall lower than 200 mm from flowering to harvest. Under these conditions it is unlikely that crops experienced severe terminal water stress, which would constitute evidence against the advantage of a high TE. More research should be carried out to elucidate this.

Early measurement of TE is suitable for high-throughput selection, since it is feasible with most available phenotyping platforms, including those available as a commercial service. A convenient alternative to be considered is carbon isotope discrimination (CID), which is used frequently in phenotyping as an estimator of TE (Tardieu et al., 2011; Masuka et al., 2012). While CID is reliably correlated to intrinsic TE measured at leaf level (i.e., the ratio of photosynthesis to stomatal conductance), it has frequently been shown to be only poorly correlated to whole-plant TE (i.e., the ratio of biomass to transpiration in a plant) in different species (e.g., Hammer et al., 1997; Krishnamurthy et al., 2007; Turner et al., 2007; Devi et al., 2011; Adiredjo et al., 2014; Velázquez et al., 2017). More research should be performed to elucidate whether CID could be used as an alternative to whole-plant biomass and transpiration measurements in soybean.

The conditions proposed by Edmeades et al. (1998) for a suitable “secondary” trait for breeding include having a positive correlation to yield. Drought tolerance in the greenhouse and in the field were used as the target traits for secondary trait selection and validation, respectively. In field data, a positive correlation was found between drought tolerance and yield in water-limited environments (i.e., negative correlation between DSI and yield). When looking at only the 7 cultivars used in Experiment 3, however, this correlation is not evident (*r* = 0.08, *p* = 0.86). Likewise, TE at 13 DAE is also not correlated with yield under dry conditions (*r* = −0.29, *p* = 0.52). The whole dataset also showed a negative correlation between drought tolerance and yield in environments with high water availability. These results suggest that selecting for high TE to improve DSI of yield could be detrimental to potential yield; performing selection based on both high TE and high potential yield could help overcome this limitation. As suggested by Blum (2009), besides selecting for traits that improve water use efficiency (such as TE), final yield and traits that improve water uptake should also be taken into account in a breeding program aiming at improving yield and yield stability in water-limited environments.

Despite an initial trend in plant phenomics toward increasingly sophisticated platforms, in recent years efforts have been made toward lowering the initial cost of hardware (e.g., Pereyra-Irujo et al., 2012; Minervini et al., 2017) and the development of open-source software (e.g., Hartmann et al., 2011; Klukas et al., 2014). But not only initial costs are a limitation; operational costs of these platforms can also be significant, especially in relation to the value of the results obtained. To the best of our knowledge, this is the first report in the literature of an analysis framework for assessing the efficiency of plant phenotyping in an automated platform. The framework used in this work could be applied for selecting the most efficient traits not only when using a phenotyping platform located in the greenhouse but also for phenotyping in other situations (e.g., in the field by applying automated or traditional phenotyping). In this work, it was assumed that the time and space allocation in a phenotyping platform constitute the main cost of phenotyping, but other factors could be considered (e.g., the cost of a specific measurement), as well as the threshold values for each criteria (e.g., repeatability).

## Conclusion

The framework of analysis used in this work proved to be useful for selecting the most efficient trait for phenotyping using an automated phenotyping platform, in order to reduce costs, time and effort. Transpiration efficiency under well-watered conditions was found to be an early and efficient trait that correlated to water deficit tolerance both under greenhouse and field conditions, and could be potentially useful for developing soybean genotypes tolerant to water deficit using automated phenotyping techniques.

## Author contributions

LA: coordinated the whole writing of the manuscript; GP and LA: conceived and designed the experiments; LP: performed the experiments, measurements, and data analysis; LP and LA: discussed the results; LP, LA, and GP: wrote the manuscript; AB and IE: performed leaf area and transpirations measurements during Experiment 3.

### Conflict of interest statement

The authors declare that the research was conducted in the absence of any commercial or financial relationships that could be construed as a potential conflict of interest.

## Footnotes

^1^Graphpad Software Inc. San Diego, CA. Available online at: http://www.graphpad.com/

## Supplementary material

Supplementary Table 1 Phenotypic traits evaluated during the Experiment 1 with GlyPh and four criteria considered for the evaluation of phenotyping efficiency.

Supplementary Table 2 Characteristics of the 80 environments evaluated from the Argentinean National Trial Network of Soybean cultivars (RECSO) database.

Supplementary Figure 1 Daily course of maximum and minimum values of temperature (°C), relative humidity (RH, %) and irradiation^**^ (μmol/m2s ^*^10) for experiment 1 **(A)**, experiment 2 **(B)**, and experiment 3 **(C)** in greenhouse during 57, 50, and 28 days after emergence (DAE), respectively. ^**^Minimum values for irradiation were near zero for all experiments (data not shown).

Supplementary Figure 2 Average yield of the seven genotypes evaluated in dry **(A)** and wet **(B)** conditions in the field.
