# Supplementary figures and images for: Assessing the Efficiency of Phenotyping Early Traits in a Greenhouse Automated Platform for Predicting Drought Tolerance of Soybean in the Field
Source: Front Plant Sci. 2018 May 3;9:587. doi: 10.3389/fpls.2018.00587 (PMC5943574; doi:10.3389/fpls.2018.00587)

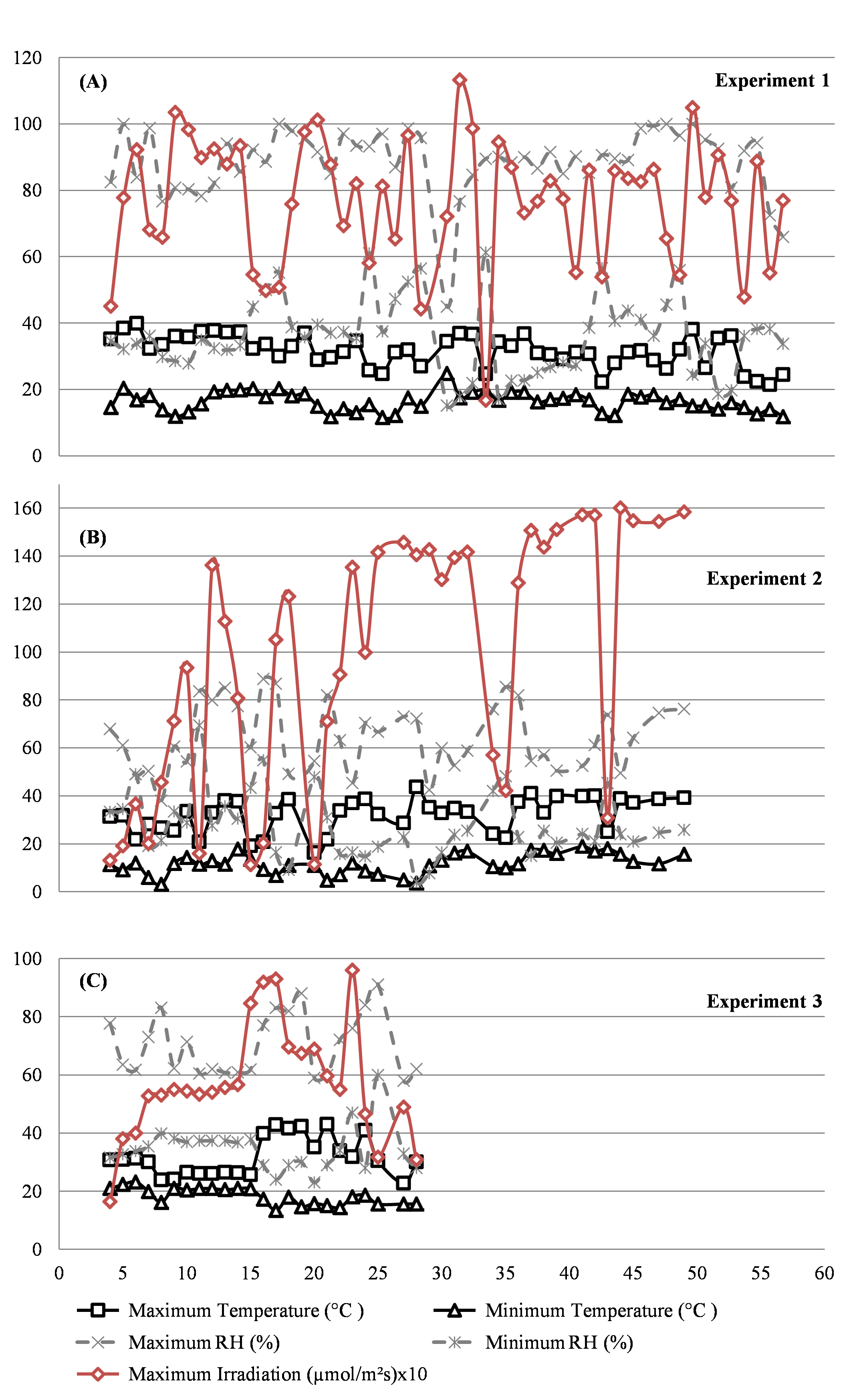

Supplement: Supplementary Figure 1 — Daily course of maximum and minimum values of temperature (°C), relative humidity (RH, %) and irradiation** (μmol/m2s *10) for experiment 1 (A), experiment 2 (B), and experiment 3 (C) in greenhouse during 57, 50, and 28 days after emergence (DAE), respectively. **Minimum values for irradiation were near zero for all experiments (data not shown). [file Image_1.TIF]

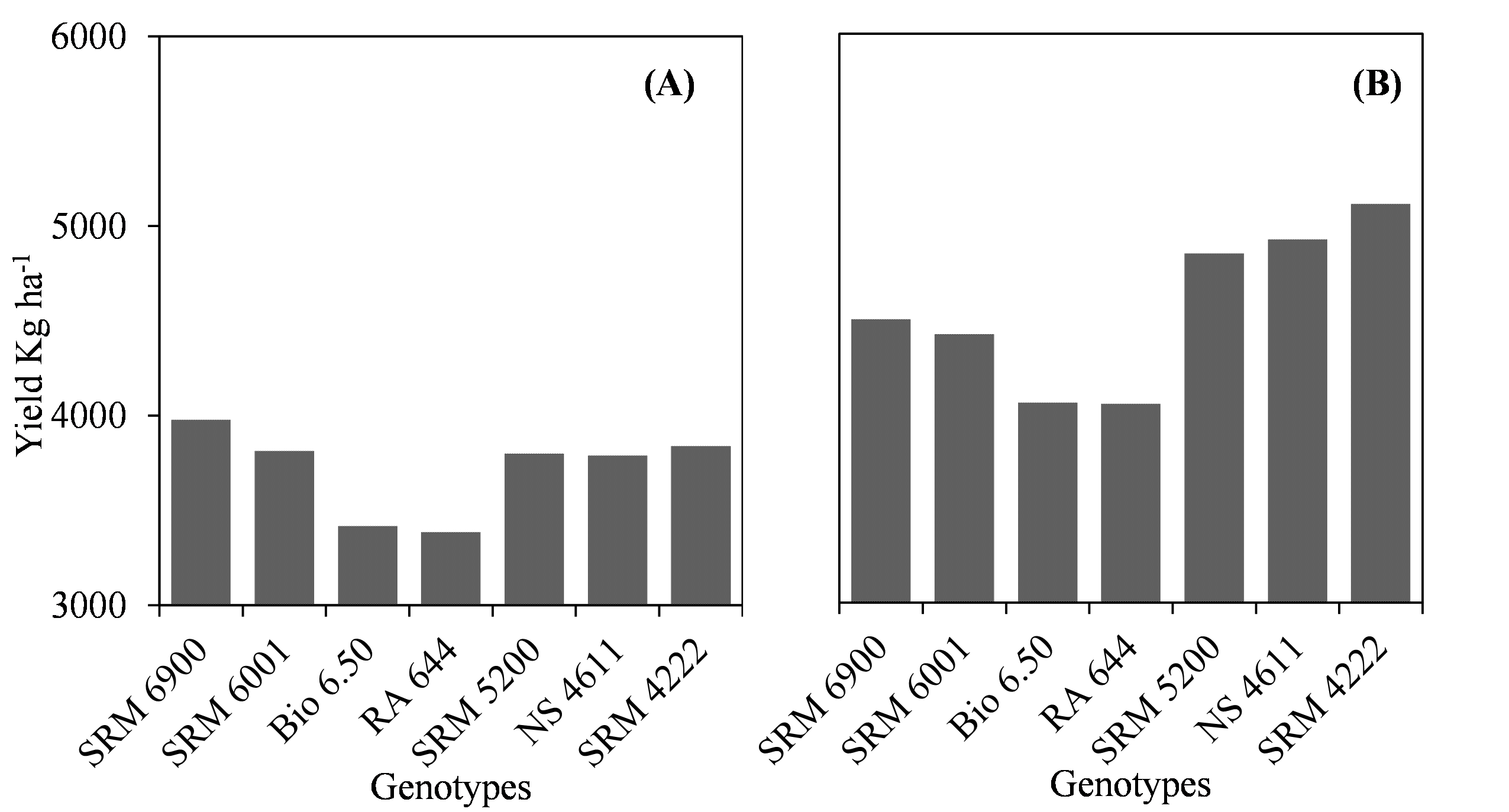

Supplement: Supplementary Figure 2 — Average yield of the seven genotypes evaluated in dry (A) and wet (B) conditions in the field. [file Image_2.TIF]
